# *Chimonanthus nitens Oliv* Polysaccharides Modulate Immunity and Gut Microbiota in Immunocompromised Mice

**DOI:** 10.1155/2023/6208680

**Published:** 2023-02-15

**Authors:** Yang Zhang, Yantian Tang, Lei Cai, Jing He, Lingli Chen, Kehui Ouyang, Wenjun Wang

**Affiliations:** ^1^Jiangxi Province Key Laboratory of Animal Nutrition, College of Animal Science and Technology, Jiangxi Agricultural University, Nanchang 330045, China; ^2^Jiangxi Key Laboratory of Natural Products and Functional Food, College of Food Science and Engineering, Jiangxi Agricultural University, Nanchang 330045, China

## Abstract

To investigate the immunomodulatory activities of *Chimonanthus nitens Oliv* polysaccharides (COP1), an immunosuppressive mouse model was generated by cyclophosphamide (CY) administration and then treated with COP1. The results demonstrated that COP1 ameliorated the body weight and immune organ (spleen and thymus) index of mice and improved the pathological changes of the spleen and ileum induced by CY. COP1 strongly stimulated the production of inflammatory cytokines (IL-10, IL-12, IL-17, IL-1*β*, and TNF-*α*) of the spleen and ileum by promoting the mRNA expressions. Furthermore, COP1 had immunomodulatory activity by increasing several transcription factors (JNK, ERK, and P38) in the mitogen-activated protein kinase (MAPK) signaling pathway. Related to the above immune stimulatory effects, COP1 positively affected the production of short-chain fatty acids (SCFAs) and the expression of ileum tight junction (TJ) protein (ZO-1, Occludin-1, and Claudin-1), upregulated the level of secretory immunoglobulin A (SIgA) in the ileum and microbiota diversity and composition, and improved intestinal barrier function. This study suggests that COP1 may provide an alternative strategy for alleviating chemotherapy-induced immunosuppression.

## 1. Introduction

As an oxazaphosphorine-substituted nitrogen mustard alkylating agent, CY is often used in clinical chemotherapy, but long-term use of CY has adverse side effects, including leukopenia, immunosuppression, increased risk of infections, and myelosuppression [1]. Immunosuppression is one of the side effects of CY, and patients are susceptible to invasive fungal infections, which can be life-threatening [2]. In recent years, many naturally active substances with low toxic effects on the host have been proven to repair the immune deficiency induced by CY [3–6].

The immune system is made up of immune organs, immune cells, and immune molecules; it refers to the body's resistance to harmful substances such as foreign bacteria and viruses. It plays a vital role in resisting infection, repairing damaged tissues, and maintaining host health [7, 8]. The intestinal tract is a metabolically active organ with unique immune function. It is the largest bacterial reservoir in the body and plays an important role in the body's digestion, absorption, metabolism, immunity, and other biological activities [9]. Intestinal microbiota has been called the “forgotten organ,” carrying the body's “second gene” [10]. In the mammalian gut, microbes can promote the maturation of the immune system and play a key role in maintaining the dynamic balance of the intestinal tract by regulating the immune response and protecting the epithelial barrier [11, 12]. In addition, the gut microbiota can activate key enzymes and metabolic pathways to metabolize complex carbohydrates and produce SCFAs, which play an important role in acidification and maintenance of intestinal homeostasis and in timely immune regulation [13–15]. The intestinal microbiota structure disorder caused by different factors will affect the body health and even cause disease. Existing studies have shown that diseases related to intestinal flora and metabolism include obesity [16, 17], insulin resistance [18], autoimmune diseases [19], and tumors [20, 21]. In recent years, a large number of studies have shown that natural polysaccharides can activate the body immunity, which slowed down the immunosuppressant effect of CY by improving the composition of intestinal microflora and enhancing intestinal barrier function [22–25].

Dried leaves of *Chimonanthus nitens Oliv* are often called golden tea, which is often used as a drink, and it often appears as a natural plant drink in the lives of local people of Jiangxi, Anhui, and Zhejiang Province; it is also known as one of the four famous drugs in Jiangxi [26]. It contains mainly polysaccharides, volatile oil, alkaloids, and flavonoids, which have many functions such as antioxidant, immune regulation, lowering blood lipid, protecting the liver, and antitumor [27]. However, there are few reports of research on the role of polysaccharides from *Chimonanthus nitens Oliv* leaves in intestinal immunity, and the underlying mechanism between this kind of polysaccharides and the immune system remains unclear. Therefore, this study selected COP1 with high purity and antioxidant properties to identify its immunomodulatory activity. This work may contribute to the further development of COP1 as an immunomodulator supplement for immunity and health.

## 2. Materials and Methods

### 2.1. Materials

Raw material was purchased from Ganzhou, Jiangxi Province, China. The SIgA diagnostic kit and the total protein assay kit (BCA method) were obtained from Nanjing Jiancheng Bioengineering Institute (Nanjing, China). The kits for the cytokines (TNF-*α*, IL-1*β*, IL-17, IL-12, and IL-10) were purchased from Wuhan Boster Biological Technology. Standard SCFAs and CY were purchased from Aladdin (Shanghai, China). Levamisole hydrochloride was purchased from Yuanye Bio-Technology (Shanghai, China). Other pure reagents for analysis were obtained locally in China.

### 2.2. Preparation of COP1

COP1 was obtained in the same way as previously reported [28]. In general, *Chimonanthus nitens Oliv* leaves were dried, crushed and sifted, soaked in petroleum ether to remove the fat soluble substance in the powder, and dried for use. Distilled water was added to the powder at 20 : 1 (*v*/*w*) and extracted for 2 h in the ultrasonic cycle at 85°C. The filtrate was collected and concentrated with a rotary evaporator. Four volumes of 95% ethanol were added to the concentrated liquid, and precipitates were collected. The precipitate was dissolved in distilled water, the alcohol was removed, and the protein was removed by Sevag method. Sevag reagent (chloroform : n-butanol = 4 : 1, *v*/*v*) was mixed with polysaccharide solution (3 : 1, *v*/*v*), shaken for 20 min, and then centrifuged. The concentrated polysaccharide solution was depressurized in vacuum and freeze-dried to obtain crude polysaccharides (COP). COP was adsorbed by DEAE cellulose column and eluted with 0.1 M NaCl solution. The eluent was collected, and the component was named COP1 after being dialyzed with tap water for 48 h, distilled water for 24 h, concentration, and lyophilization. Its carbohydrate, uronic acid, and protein contents were 83.15 ± 1.17%, 10.36 ± 0.54%, and 1.61 ± 0.36%, respectively. COP1 was 18.843 kDa and mainly consisted of Glu, Xyl, Gla, and Ara, with percentage of moles 7.4 : 11.1 : 24.9 : 56.6 [28].

### 2.3. Experiments on Animals

**Figure 1:**
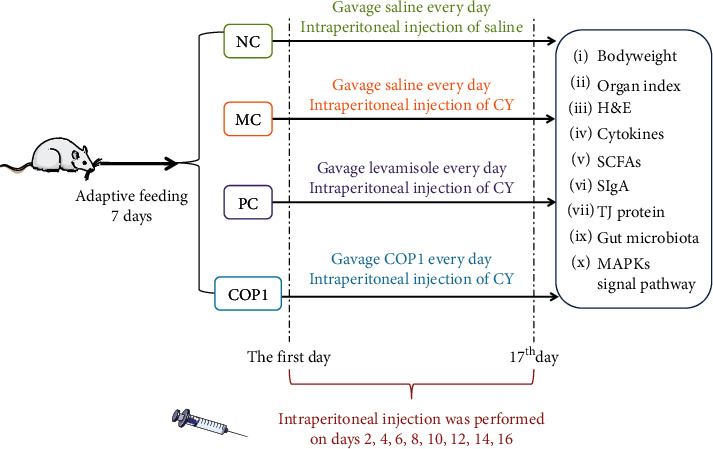
Workflow of the experiment procedure: (1)–(10) were performed for each group.

Hunan Silaike Laboratory Animal Co., Ltd. (SCXK (Xiang) 2016-0002, Changsha, China) provided ICR female mice weighing 20.0 ± 2.0 g, 6–8 weeks old. As shown in Figure 1, after a week of adaptive feeding, the mice were divided into 6 groups with 10 mice in each group: the normal group (NC), the model group (MC), the positive group (PC), and the low-, medium-, and high-dose polysaccharide groups (LCOP1, MCOP1, and HCOP1), and they were free to access the feed and water. The NC and MC were gavage normal saline, PC was gavage 40 mg/kg levamisole, and COP1 low, medium, and high doses were gavage 150, 300, and 600 mg/kg COP1, respectively. The NC was intraperitoneally injected with normal saline, and the other groups were intraperitoneally injected with CY. The body weight of the mice was recorded every three days during the experiment, and the mice were killed by humanitarian methods after the end of the experiment. The organs of mice were washed with cold normal saline and stored at −80°C. The contents of the cecum were treated with liquid nitrogen and stored to −80°C.

#### 2.3.1. Determination Index of Immune Organs and Hematoxylin and Eosin (H&E) Staining of the Spleen and Ileum

After weighing immune organs (spleen and thymus), the organ index of mice was calculated according to the following formula:

(1) $$\begin{array}{c} \text{thymus index}=\frac{\text{thymus weight}\left(\text{mg}\right)}{\text{body weight}\left(\text{g}\right)},\\ \text{spleen index}=\frac{\text{spleen weight}\left(\text{mg}\right)}{\text{body weight}\left(\text{g}\right)}. \end{array}$$

The spleen and ileum were fixed with 4% paraformaldehyde solution. H&E staining was used to observe histopathological changes.

#### 2.3.2. Measurement of Cytokines in Spleen and Ileum Tissue

Spleen and ileum tissues were collected, and phosphate buffer solution (PBS) was added at a ratio of 10 : 1, homogenized at 4°C, and centrifuged for 15 min (4°C, 10,000 rpm), and the supernatant was stored for use. The production of TNF-*α*, IL-1*β*, IL-12, IL-17, and IL-10 in the supernatant of intestinal and spleen tissues and the level of SIgA in the supernatant of intestinal tissues were determined by commercial ELISA kits. The protein content of the supernatant of spleen and ileum tissue was determined by the BCA protein assay kit.

#### 2.3.3. Determination of SCFAs in Cecal Content

SCFAs: acetic acid, propionic acid, and butyric acid, were evaluated by the reported method [29]. The organic phase was taken out and analyzed using a GC (Shimadzu GC-2010 system, Shimadzu, Kyoto, Japan) equipped with a capillary column of DB-WAX (30.0 m × 0.32 mm × 0.25 *μ*m Agilent Co., Ltd, USA). Inlet temperature is 250°C, split ratio is 10 : 1, sample volume is 1 *μ*L, carrier gas flow is 1.2 mL/min, programmed temperature to 120°C, at the rate of 5 degrees Celsius per minute to 180°C, hold for 1 min, detector (FID) 260°C, hydrogen flow: 40 mL/min, air flow: 400 mL/min, tail blowing flow 30 mL/min. The content of SCFAs was calculated based on the external standard method.

#### 2.3.4. RNA Extraction and Real-Time Quantitative Polymerase Chain Reaction (RT-qPCR) Analysis

The process of detecting mRNA levels of TNF-*α*, IL-1*β*, IL-12, IL-17, IL-10, Claudin-1, Occludin-1, and ZO-1 was conducted as the previous method [28]. Total RNA was prepared from 20 mg spleen and ileum tissues by the RNA preparation kit (TransGen Biotech, China), and RNA concentration and purity were detected by NanoDrop 2000 (Thermo Fisher Scientific Inc., USA). The mRNA was then reverse-transcribed into cDNA using a reverse transcription kit (TransGen Biotech, China) and amplified by RT-qPCR. RT-qPCR system was 20 *μ*L, consisting of 2 *μ*L cDNA, 10 *μ*L Taq PCR super mix containing SYBR Green I dye, 0.4 *μ*L forward and reverse primers, and 7.2 *μ*L nuclease-free water. The process includes the following conditions: initial incubation at 94°C for 30 s, 42 cycles including denaturation at 94°C for 30 s, annealing at 62°C for 15 s, and extension at 70°C for 30 s. In this study, the 2 ^−△△CT^ method was used to calculate gene expression.

#### 2.3.5. Western Blot Assay

The ileum tissue was crushed in RIPA lysate containing PMSF and centrifuged in an ice bath. Loading buffer was added to the supernatant, and the denaturation was boiled in boiling water. After electrophoresis, the protein was transferred onto polyvinylidene difluoride membrane (PVDF, Millipore Co., MA, USA). Then, monoclonal antibodies were matched (antibody of Claudin-1, Occludin-1, ZO-1, JNK/p-JNK, p38/p-p38, and ERK/p-ERK); dilution ratio of all primary antibodies and secondary antibodies is shown in Supplementary Table S1. Blots were visualized using enhanced tetrahydrochloric acid chemiluminescence (ECL) and imaged with a Gene Genius Bioimaging System (SYNGENE Co., MD, USA).

#### 2.3.6. High-Throughput Sequencing and Bioinformatics Analysis

The cecum contents of 4 mice in each group were randomly selected, and genomic DNA was extracted using E.Z.N.A. ® Soil DNA Kit (Omega Bio-Tek, Norcross, GA, U.S.) according to the manufacturer's instructions and stored at −80°C for 16S ribosomal RNA (rRNA) gene amplification sequence determination. The V3-V4 regions of bacterial 16S rRNA were selected for amplification and then sequenced by an Illumina MiSeq platform (USA). The data were used for further bioinformatics analysis. The sequence data were stored in the NCBI Sequence Read Archive (SRA) database and analyzed on the free online platform of Majorbio Cloud Platform. The *α*- and *β*-diversity analysis, principal component analysis (PCA), linear discriminant analysis effect size (LEfSe), and linear discriminant analysis (LDA) were performed using the free online platform of Majorbio Cloud Platform (http://www.majorbio.com).

### 2.4. Statistical Analysis

Data analysis was performed with SPSS software, and the Duncan method was used for significance analysis. All results were presented as mean ± standard deviation (*M* ± SD).

## 3. Results

### 3.1. COP1 Restored the Body Weight and Immune Organ Indexes

Table 1 shows the changes in the average body weight of the mice during the experiment. After CY intervention, the body weight of MC increased briefly and then decreased continuously, which was significantly lower than that of NC indicating successful modeling. The body weight of mice in PC and COP1 recovered obviously, and the effects of MCOP1 and HCOP1 were similar to those of PC. These results indicated that long-term high-dose COP1 could alleviate the weight loss caused by CY.

Compared with NC, the spleen and thymus indexes of MC were significantly decreased (Table 2) (*p* < 0.01). After gavage of COP1 at different doses, the spleen and thymus indexes were significantly increased (*p* < 0.01), and the immune organ indexes were restored to normal level in the MCOP1 and HCOP1. These results indicated that COP1 could enhance spleen index and thymus function in CY-treated mice to a certain extent.

### 3.2. COP1 Restored the Histological Changes of the Spleen and Ileum

**Figure 2:**
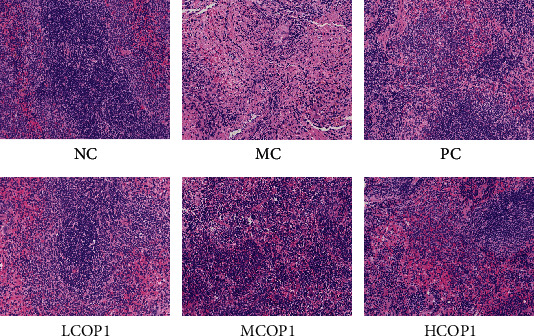
Histopathology observation of the spleen, original magnification: ×200.

H&E staining results showed that the spleen structure (Figure 2) of NC was clear, lymphocytes were closely arranged, the structure of red and white pulp was regular, and the edge was clear. In MC, the white pulp was blurred, the area was smaller, and the number of lymphocytes was sparse and decreased. COP1 alleviated the damage of CY to spleen tissue, and both MCOP1 and HCOP1 have a positive effect.

**Figure 3:**
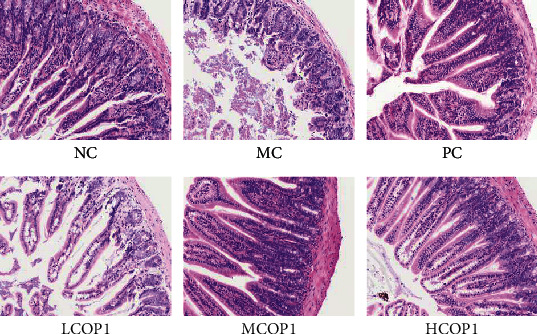
Histopathology observation of the ileum, original magnification: ×200.

Under a light microscope, ileum villi in NC were complete and closely arranged (Figure 3). In the ileum villus section of MC, incomplete structure, thinning muscular layer, short villi, sparse, irregular arrangement, and other serious lesions were observed. The histological morphology of the mouse ileum was improved by gavage of COP1.

### 3.3. COP1 Upregulated the Cytokine Level and Related Gene Expression of the Spleen and Ileum

**Figure 4:**
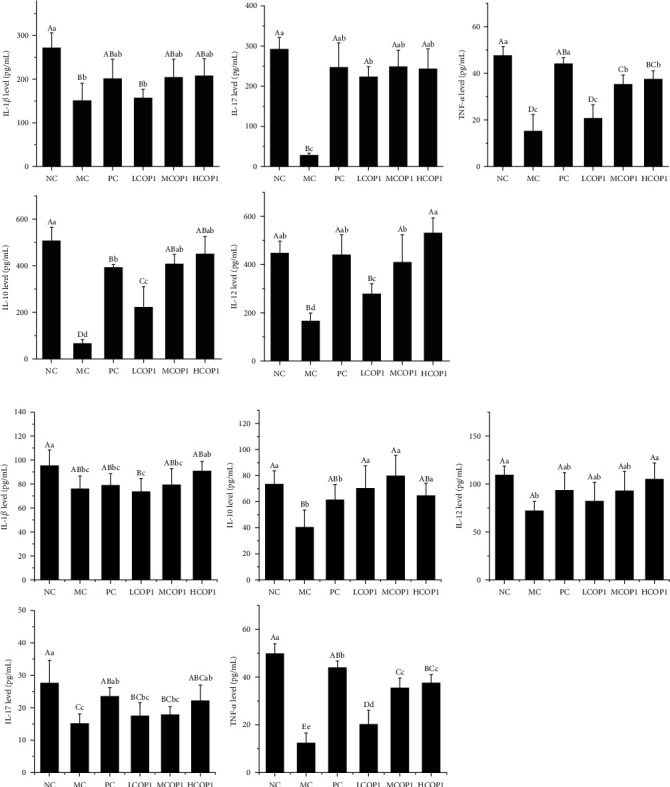
Cytokine level of the (a) ileum and (b) spleen in different groups. Animals were sacrificed to harvest the spleen that was subjected to determine TNF-*α*, IL-1*β*, IL-17, IL-12, and IL-10 content. The indicators were normalized with total protein content; the values were presented as mean ± SD (*n* = 6). On each measure, the difference in letters between the groups indicated significant differences by the letters abcd (*p* < 0.05) and ABCD (*p* < 0.01). Duncan analysis was used for data variance analysis.

**Figure 5:**
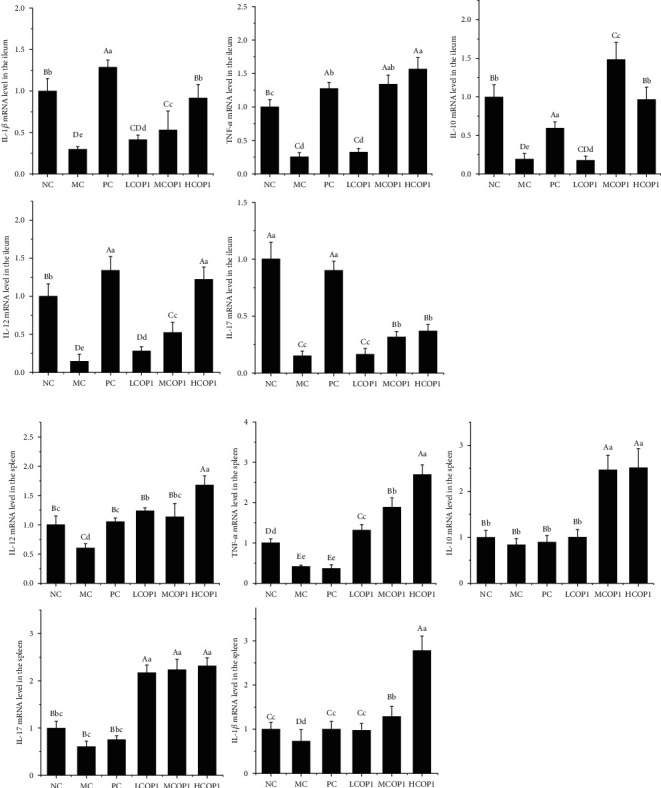
Cytokines relate mRNA level of the (a) ileum and (b) spleen in different groups (*n* = 6). Animals were sacrificed to harvest the spleen that was subjected to determine TNF-*α*, IL-1*β*, IL-17, IL-12, and IL-10 content. On each measure, the difference in letters between the groups indicated significant differences by the letters abcd (*p* < 0.05) and ABCD (*p* < 0.01). Duncan analysis was used for data variance analysis.

Figure 4 shows the contents of inflammatory factors in spleen tissue and ileum tissue of mice in each group, respectively. CY treatment reduced the contents of inflammatory factors in the spleen and intestinal tissue of mice, and COP1 restored the contents of inflammatory factors, and the effect of MCOP1 and HCOP1 is better than that of LCOP1. In spleen tissue, the levels of TNF-*α*, IL-10, and IL-17 were significantly increased in the MCOP1 and HCOP1, and the level of IL-12 was restored to the NC in the HCOP1. In the ileum, the levels of IL-10, IL-17, IL-12, TNF-*α*, and IL-1*β* in three COP1 dose groups were significantly increased (*p* < 0.01), and the effect of high-dose COP1 on the secretion of inflammatory cytokines is similar to that of positive drugs. The mRNA expression of inflammatory cytokines in the spleen and ileum showed the same trend as secretion of inflammatory cytokines in tissues (Figure 5); CY decreased the mRNA expression of each inflammatory factor, while COP1 increased the mRNA expression. It suggests that COP1 promoted the secretion of inflammatory cytokines in the spleen and ileum by promoting cytokine mRNA expression.

### 3.4. COP1 Regulated Immunity by Regulating the MAPK Pathway

**Figure 6:**
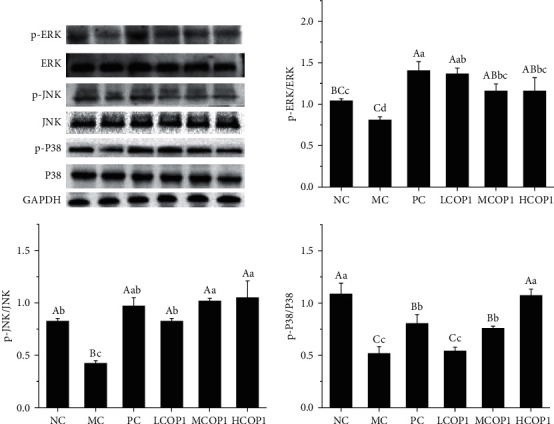
Effect of COP1 on the phosphorylation of mitogen-activated protein kinase (MAPK) in splenocytes of CY-treated mice (*n* = 3). On each measure, the difference in letters between the groups indicated significant differences by the letters abcd (*p* < 0.05) and ABCD (*p* < 0.01). Duncan analysis was used for data variance analysis.

In order to further investigate the mechanisms underlying the immunomodulatory effects of COP1, we examined the phosphorylation levels of JNK, ERK, and P38 proteins in the MAPK pathway (Figure 6). The ratios of p-JNK/JNK, p-ERK/ERK, and p-p38/p38 in ileum tissue of the MC were significantly decreased (*p* < 0.01). Compared with MC, the phosphorylation levels were upregulated in MCOP1 and HCOP1. It is proved that COP1 can alleviate immune deficiency by activating the MAPK signaling pathway.

### 3.5. COP1 Promoted SCFA Level of Immunocompromised Mice

**Figure 7:**
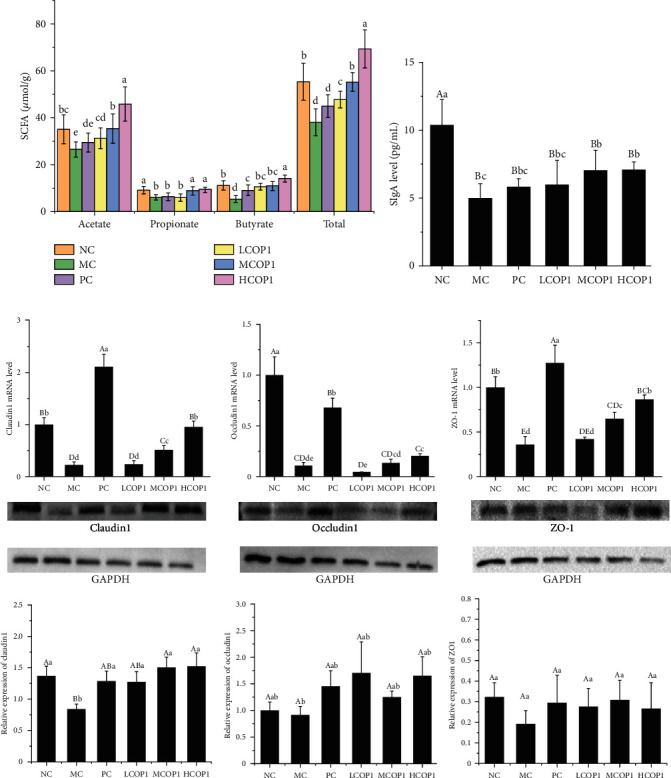
(a) Short-chain fatty acid content in cecal contents of different groups of mice (*n* = 6). (b) SIgA level in the ileum of mice (*n* = 6). (c) The mRNA and TJ protein expression of Claudin-1, Occludin-1, and ZO-1 in different groups (*n* = 3). On each measure, the difference in letters between the groups indicated significant differences by the letters abcd (*p* < 0.05) and ABCD. Duncan analysis was used for data variance analysis.

Fermentation is an important function of intestinal microorganisms, and most SCFAs are acetic acid, propionic acid, and butyric acid (90–95%) [30]. Therefore, the levels of three fatty acids, acetic acid, propionic acid, and butyric acid, in cecum contents were detected in this experiment. Compared with NC, the levels of acetic acid, propionic acid, and butyric acid in MC were significantly decreased (*p* < 0.05, Figure 7(a)). The levels of acetic acid, butyric acid, and total SCFAs were significantly increased in the three COP1 treatment groups compared with MC, and the levels of propionic acid in MCOP1 and HCOP1 were also significantly increased (*p* < 0.05). In addition, the enhancement effect of SCFAs in MCOP1 and HCOP1 was better than that in PC and LCOP1, the contents of acetic acid, propionic acid, butyric acid, and total SCFAs in MCOP1 recovered to the level of NC group, and the contents of three kinds of SCFA and total SCFAs in HCOP1 exceeded those in the NC group.

### 3.6. COP1 Upregulated the Expression of SIgA and TJ Protein Level

SIgA levels in ileum tissue of mice in different groups are shown in Figure 7(b). The content of SIgA in the ileum mucosa of MC was significantly lower than that in NC. Compared with the MC, the content of the mice in MCOP1 and HCOP1 significantly increased, and the recovery effect was more than that of PC (*p* < 0.05).

The expression of TJ proteins (ZO-1, Claudin-1, and Occludin-1) can indicate intestinal barrier function. Therefore, the mRNA and protein expression levels of TJ proteins were evaluated (Figure 7(c)). Both positive drug and COP1 could significantly upregulate the expression of ZO-1, Occludin-1, and Claudin-1 (*p* < 0.01). These results suggest that COP1 restored intestinal mucosal barrier function by promoting the expression of TJ protein and the secretion of SIgA in immunosuppressed mice.

### 3.7. High-Throughput Sequencing and Bioinformatics Analysis

**Figure 8:**
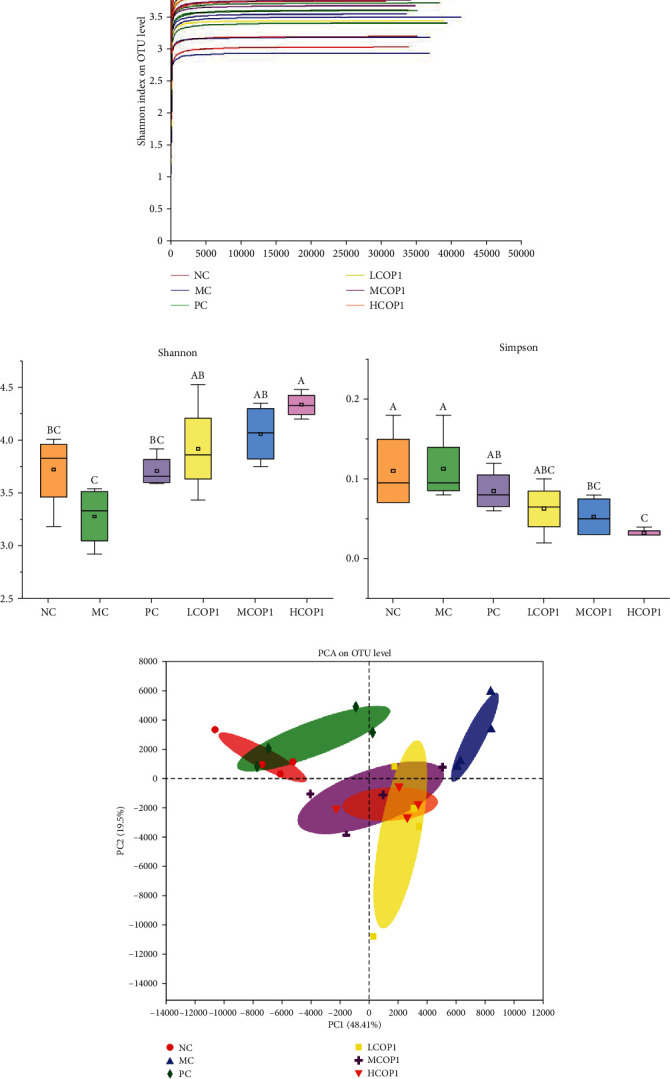
Effect of COP1 on gut microbiota composition. (a) Shannon-Wiener curve; (b) Shannon index; (c) Simpson index; (d) principal component analysis (PCA) of gut microbiota in different groups. On each measure, the difference in letters between the groups indicated significant differences by the letters abcd (*p* < 0.05) (*n* = 4). Duncan analysis was used for data variance analysis.

The gut microbiota alteration induced by COP1 was evaluated by 16S rRNA gene sequencing of the samples isolated from mouse cecum. With the increase of sequencing volume, the number of newly added operational taxonomic units (OTUs) gradually decreases; the Shannon-Wiener curve (Figure 8(a)) was flat, indicating that the measured data are saturated and can reflect the information of most of the bacterial communities in the sample. Shannon and Simpson diversity indices (Figures 8(b) and 8(c)) were calculated using Tukey's test to describe the alpha diversity, which refers to the diversity within a specific region or ecosystem. After CY intervention, Shannon index decreased and Simpson index changed not significantly. Compared with MC, the Shannon index and Simpson index of HCOP1 were significantly increased (*p* < 0.05).

**Figure 9:**
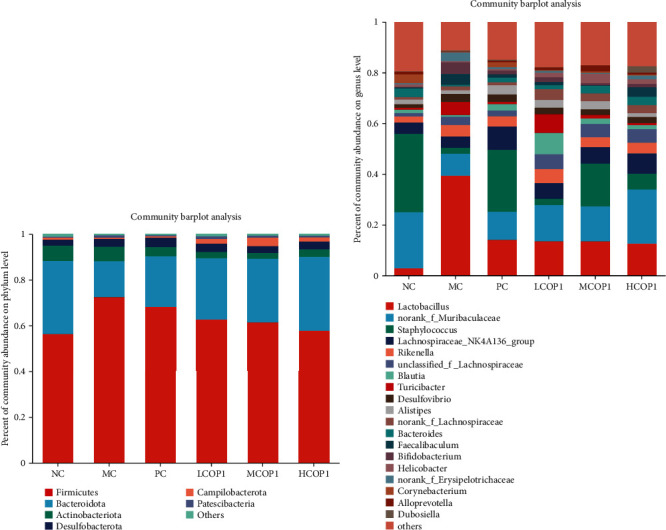
Microbial community stack column plot by (a) phylum and (b) genus, respectively (*n* = 4).

**Figure 10:**
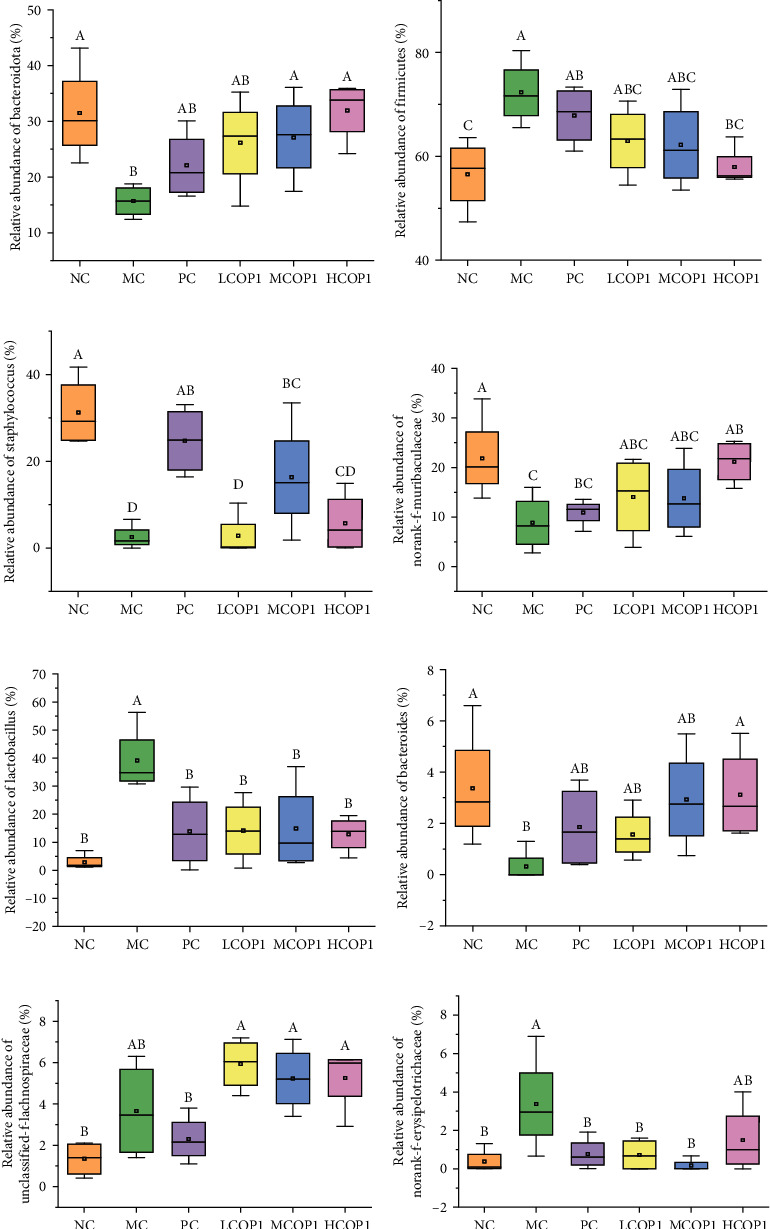
Box-and-whisker plots of 3 different abundances of gut microbiota at the phylum level. On each measure, the difference in letters between the groups indicated significant differences by the letters abcd (*p* < 0.05, *n* = 4). Duncan analysis was used for data variance analysis. (a, b) *Firmicutes* and *Bacteroidota* at the phylum level and (c–h) *Staphylococcus*, *norank-Muribaculaceae*, *Lactobacillus*, *Bacteroides*, *unclassified-f-Lachnospiraceae*, and *norank-Erysipelotrichaceae*.

The principal component analysis (PCA) was applied to portray the relationship between microbial communities for each treatment based on OTUs. The results showed that the microbial community structure of LCOP1, MCOP1, and HCOP1 was closer to that of the NC than the MC (Figure 8(d)). At the level of phylum (Figure 9(a)), CY treatment increased *Firmicutes* and decreased *Bacteroidetes*; levamisole and COP1 treatment mitigated this trend. At the genus level (Figure 9(b) and Figure 10), CY treatment significantly reduced the contents of *Staphylococcus*, *norank-Muribaculaceae*, and *Bacteroides*, while it increased the content of *Lactobacillus* and *norank*-*Erysipelotrichaceae* (*p* < 0.05). At the same time, CY also had an effect on *Turicibacter*, *Lachnospiraceae-NK4A136-group*, and *Alistipes*. COP1 intervention could reduce the effect of CY on the abundance of microbial flora.

**Figure 11:**
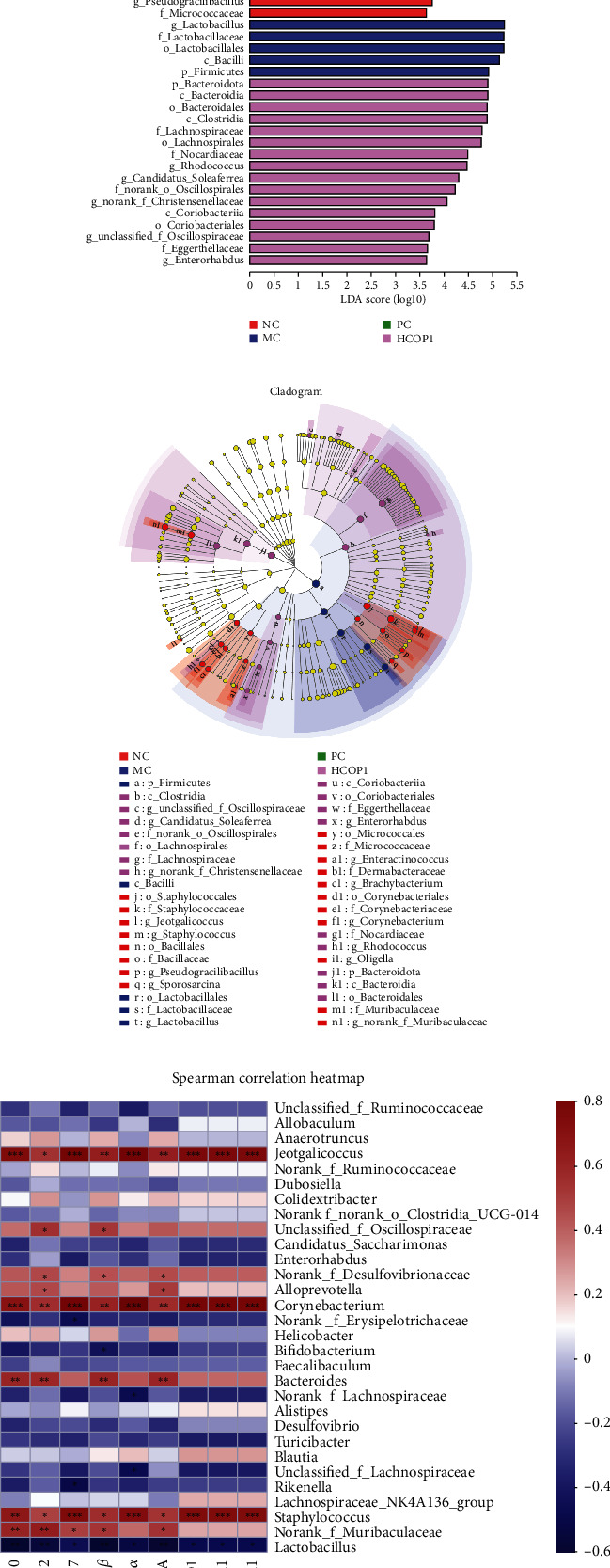
The LEfSe and Spearman correlation analyses of the NC, MC, PC, and HCOP1 groups (*n* = 4). (a) LDA score. Enriched taxa with an LDA score > 3.6 are shown in the histogram. The greater the LDA score was, the more significant the phylotype microbiota was in the comparison. (b) LEfSe taxonomic cladogram. The colored nodes from the inner circle to the outer circle represented the hierarchical relationship of all taxa from the phylum to the genus level. Taxa enriched in the COP1 group are showed in purple and in the NC group are showed in red while taxa enriched in the M group are shown in blue and taxa with nonsignificant changes are colored in white. The diameter of each small circle represented the taxa abundance. (c) The composition of gut microbiota was analyzed based on the top 30 species at the genus level showed by a heatmap.

In view of the best therapeutic effect of high-dose COP1, HCOP1 was selected as the representative of COP1 intervention groups, and further analysis was conducted with NC, MC, and PC. Linear discriminant analysis effect size (LEfSe) was used to analyze the representative species of intestinal microbiota, and nonparametric factorial Kruskal-Wallis sum rank test and linear discriminant analysis (LDA) were used to analyze significant differences in bacterial abundance. Based on LEfSe analysis, we observed 19 discriminative characteristics in NC among which the main bacterial groups were *Staphylococcus*, *Muribaculaceae*, and *Corynebacterium*. MC showed 4 dominant microorganisms; the main bacterium group was *Lactobacillus*. HCOP1 had 16 dominant flora (LDA > 3.6, *p* < 0.05), mainly *Bacteroidota* and *Lachnospiraceae*. However, according to the analysis, there were no dominant microorganisms in PC (Figure 11(a)). Then, an evolutionary cluster analysis map was given to determine the main microbiota by taxonomy. In cladogram, *Bacteroidota*, *Clostridia*, and *Coriobacteriia* in the purple part had the highest abundance, while *Bacilli* in the blue part had the highest abundance, representing HCOP1 and MC, respectively (Figure 11(b)). Overall, these results suggest that COP1 treatment altered the key phylotypes of gut microbiota in the feces of CY-treated mice and promoted the proliferation of specific bacteria.

In order to identify the gut microbiota that might contribute to modulate immunity in CY-treated mice, the correlations between the relative abundances of main gut microbiota at the genus level and parameters of immune responses were analyzed by Spearman's correlation analysis. As shown in Figure 11(c), characteristics showing that the levels of these bacteria are closely related to immunity include ileum tissue cytokines and gene expression levels of SIgA and TJ proteins. Among them, *Brachybacterium*, *Alloprevotella*, *Jeotgalicoccus*, *Corynebacterium*, *Staphylococcus*, *Bacteroides*, *norank-f-Muribaculaceae*, *unclassified-f-Oscillospiraceae*, and *norank-f-Desulfovibrionaceae* were positively correlated with immune traits, while *Lactobacillus*, *Rikenella*, *Enterorhabdus*, *Turicibacter*, and *norank-f-Erysipelotrichaceae* were negatively correlated with immune traits.

## 4. Discussion

CY is one of the most effective and widely used antitumor drug; however, long-term use of CY can cause damage to the immune system and even be life-threatening [31]. Modulating immune responses to alleviate disease has attracted great interest over the years [32]. At the same time, many polysaccharides isolated from plants, animals, and fungi have attracted much attention because of their potential immunomodulatory activities [8, 33, 34]. *Chimonanthus nitens Oliv* is one of the four famous medicines in Jiangxi Province, which has a variety of biological activities. In our previous study, a novel polysaccharide COP1 was isolated from *Chimonanthus nitens Oliv* leaves. To explore the immunomodulatory activity of COP1, CY-treated mice were used as experimental models in this study.

The spleen and thymus glands are important immune organs. The thymus can regulate peripheral immune organs and immune cells, and T cells differentiate and mature in the thymus. After stimulation, the spleen can differentiate a large number of T and B lymphocytes to participate in the body's immunity. Therefore, the changes of spleen and thymus quality can reflect the changes of the number of lymphocytes in the body and thus indirectly reflect the immune response level of the body [35, 36]. Our current results suggest that the administration of COP1 can effectively enhance thymus and spleen indicators (*p* < 0.01), suggesting that COP1 may enhance the host immune system despite promoting immune organs. Cytokines are small molecular glycoproteins synthesized and secreted by immune cells and some stromal cells. By binding to corresponding receptors, cytokines are important factors in regulating cell growth, differentiation, and immune response. For example, IL-12 can activate T cells and induce the differentiation of Th1 cells, which can promote B cell proliferation and antibody secretion and NK cell proliferation [37, 38]. TNF-*α* is an important mediator of immune protection, promoting cell proliferation and differentiation and enhancing the killing ability of killer cells such as T cells. IL-10 is generally considered as a protective cytokine that promotes immune regulation, and IL-17 is involved in host defense against fungi, extracellular bacteria, and other eukaryotes [39, 40]. Previous studies have shown that the effects of polysaccharides on improving immune function are closely associated to their ability to promote cytokine secretion [3, 41–44]. In our experiments, COP1 significantly upregulate cytokine (IL-10, IL-17, IL-12, IL-1*β*, and TNF-*α*) production of the spleen and ileum, which could help immunocompromised mice restored.

The MAPK signaling pathway is usually involved in regulating the synthesis and release of proinflammatory cytokines and the expression of related mRNA, thereby modulating the immune response [42]. Previous studies have provided us with some evidence that polysaccharides activate the MAPK signaling pathway. *G. frondosa* polysaccharides could exert immune regulation by activating the MAPK signaling pathway in macrophages and immunocompromised mice [38, 43]. *Mesona chinensis Benth* polysaccharides could protect mice from CY by upregulating the phosphorylation levels of protein factors in MAPK signal transduction pathways [44]. In this study, COP1 stimulated the activation of the immune system in immunocompromised mice via the phosphorylation of MAPKs, such as JNK, ERK, and p38. The results suggested that COP1 exhibited immunomodulatory activities by upregulating various transcription factors in MAPK signaling pathways. In addition, the upregulation of cytokine (IL-10, IL-17, IL-12, IL-1*β*, and TNF-*α*) secretion and mRNA expression may be achieved through the MAPK signaling pathway. These findings have important implications for understanding the molecular mechanisms of polysaccharides derived from *Chimonanthus nitens Oliv* leaves.

The intestinal tract is a metabolically active organ with unique immune function. The intestinal protective barrier is mainly reflected in biological barrier, immune barrier, and physical barrier. And the cytokines involved in immune barrier are affected by the MAPK signaling pathway [45]. Liu et al. and Shi et al. showed that intraperitoneal injection of CY could cause intestinal mucosal damage [34, 46]. SIgA plays an important role in intestinal mucosal immune barrier, and the content of SIgA reflects the immune function of the gut mucosa. When intestinal damage occurs, SIgA secreted by the body can slow down intestinal mucosal damage and maintain the normal operation of intestinal immune barrier function [47]. In this study, high-dose COP1 significantly promotes the secretion of SIgA in the ileum tissue of immunosuppressive mice, and its effect was consistent with that of levamisole. The function of physical barrier is determined by TJ proteins between intestinal cells, which are composed of related TJ proteins, such as atretic protein Occludin, precision connexin ZO, and sealing protein Claudin, which perform roles of promoting and maintaining cell polarity, regulating signal transduction, mediating, and adhesion, respectively [48–50]. In this study, COP1 treatment repaired pathological damage to intestinal tissue and maintained the physical barrier by enhancing the expression of TJ protein (ZO-1, Occludin-1, and Claudin-1)-related genes and proteins, thus achieving immune protection.

The intestinal tract is the largest host of most bacteria in the body, and these resident bacteria constitute a complex and huge microecosystem, known as the intestinal biological barrier [51]. This study used 16S rRNA sequencing to detect the diversity and composition of the microbiota. The analysis of *α*-diversity and *β*-diversity of gut microbiota showed that the diversity and species composition of gut microbiota changed significantly after CY treatment. Compared with MC, Shannon index and Simpson index were significantly changed after treatment of the dose of a 600 mg/kg/d COP1, which proved that the richness and evenness of intestinal microbiota in mice were improved after COP1 treatment.

*Firmicutes* and *Bacteroidetes* both possess carbohydrate-related enzymes that make use of dietary polysaccharides. *Bacteroidetes* is the main decomposition bacteria of complex carbohydrates, while *Firmicutes* prefer to use oligosaccharides [52]. COP1 reversed the CY-induced reduction in B/F index, which was similar to *Mulberry leaf*, *Lonicerae flos*, and *Lycium barbarum* polysaccharides [23, 53, 54]. *Staphylococcus* is reported to colonize the nasopharynx, skin, and gastrointestinal tract of the organism. Humans and their domesticated animals are colonized by different species of *Staphylococcus*, of which only *S. aureus* evolved to consistently cause invasive disease in healthy immunocompetent individuals [55, 56]. In this study, *Staphylococcus*, the protodominant strain in ICR mice, should be basically nonpathogenic. *Muribaculaceae* belongs to *Bacteroides*, which has been found to have probiotic effects and related to the innate immune system. It is reported that homeostatic IgA responses could target *Muribaculaceae* residing in the small intestine [57]. In addition, *Muribaculaceae* has the function of degrading complex carbohydrates and is positively correlated with the concentrations of intestinal acetate, propionate, and butyrate [58]. *Bacteroides*, an abundant genus of bacteria colonized in the intestinal tract of mammals, has been recently considered as a candidate for the next generation of probiotics because of its potential to promote host health. It can stimulate the immune system, enhance the phagocytosis function of macrophages, and resist the colonization of pathogenic bacteria to promote host health [59]. In addition, *Bacteroides* and *Lachnospiraceae* are major players in the synthesis of SCFA in the gut, and groups of bacteria *Lachnospiraceae* and *Muribaculaceae* possess the ability to produce butyric acid by enzyme [60, 61]. Some *Turicibacter* bacteria have a pathogenic lifestyle. To date, typical strains of *Turicibacter* have been isolated from male patients with acute appendicitis with fever and from elderly women with fever [62]. In addition, it can lead to some members of *Muribaculaceae* associated with cluster ecological disorder [63, 64]. *Erysipelotrichaceae* is commonly found in the intestinal cavity where gastrointestinal diseases occur. Chen and Zhu et al. observed an increased abundance of *Erysipelotrichaceae* in patients with colorectal cancer and in animal models of colon cancer, respectively [65, 66]. In addition, enrichment of *Erysipelotrichaceae* was found in obese and liver damaged mice [67, 68]. Coincidentally, it was found in previous studies that CY caused liver damage in mice, and the enrichment of *norank-Erysipelotrichaceae* in this experiment suggested the pathological environment in the intestinal tract of mice [28]. It should be noted that CY treatment significantly increased the content of *Lactobacillus*, which was not the dominant genus of intestinal microorganisms in the normal mice, and COP1 decreased its abundance. Although most *Lactobacillus* species are probiotics, high levels of *Lactobacillus* may do not have a beneficial effect on intestinal health. *Lactobacillus* enrichment was also observed in studies of mice receiving high doses of immunosuppressant with concanavalin A and chemotherapy with paclitaxel. The intestinal permeability of the former mice increased, while the bacterial mix in the intestinal microbiome of the latter mice changed and reduced the diversity of fecal bacteria, suggesting that the interaction between the immune system and *Lactobacillus* may be conservative [69, 70]. In addition, there are reports that the significantly increased abundance of *Lactobacillus* in small bowel transplant, surgical patients, chronic spontaneous urticaria patients, and diabetic patients was also observed [71–74]. Other studies have found that high-calorie diet causes significant concentrations of *Lactobacillus*, resulting in gut microbial imbalance and lipid metabolism disorders [75]. However, it cannot be ruled out that the increase of lactic acid bacteria is a kind of self-protection reaction against adverse environment. In this study, CY caused biased constitution in the intestinal microbial community structure of the sick organism, and COP1 intake increased the relative abundance of *Muribaculaceae*, *Bacteroides*, and *Lachnospiraceae* and decreased the relative abundance of *Turicibacter*, Lactobacillus, and norank-*Erysipelotrichaceae*.

**Figure 12:**
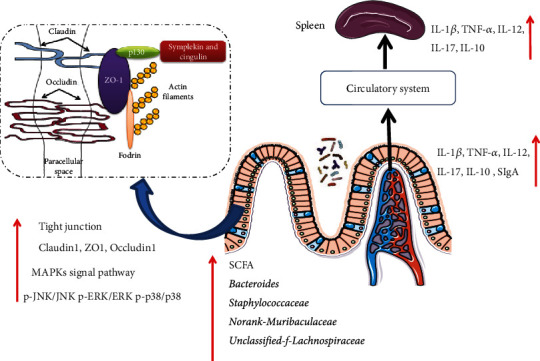
Possible mechanism of COP1 activating immunity in mice.

SCFAs as the main end products of intestinal microbial fermentation of complex carbohydrates, mainly composed of acetate, propionate, and butyrate, have been estimated to provide approximately 60–70% of the energy requirements of colonic epithelial cells [76, 77]. They can regulate the function of the innate and adaptive immune systems, and when they enter the circulatory system, they can influence the metabolism and function of surrounding tissues [78, 79]. Previous studies have shown that the increase in acetic and butyric acid production caused by polysaccharides was due to the fermentation of glucuronic acid, galactose, and xylose, while the release of propionic acid was due to the fermentation of arabinose, glucose, and xylose [80–82]. COP1 are composed of arabinose, galactose, glucose, and xylose, and the HCOP1 group has the highest content of acetate, propionate, and butyric acid, so one reason for the increase in SCFAs is the intake of COP1. In addition, another reason for the increase in SCFA may be that COP1 intervention increases the abundance of SCFA-producing bacteria mentioned above. As reported, gut microbes and their metabolites can act on or trigger intestinal mucosal immunity and then modulate the homing response of immune lymphocytes and cytokines throughout the body, as cytokines and immunoglobulins migrate from the intestine to the splenic mucosal immune system and surrounding lymphocytes to exert its systemic immune response [83, 84]. Therefore, as shown in Figure 12, the increase of cytokine content in spleen tissue should be the result of the activation of intestinal immunity. Overall, these findings may provide a basis for using COP1 as an effective immunopotentiating therapy or an alternative strategy in lessening chemotherapy-induced immunosuppression.

## 5. Conclusion

In conclusion, the present study demonstrated that COP1 could activate the immune system and regulate intestinal microbiota in immunocompromised mice. The results suggested that COP1 could effectively protect CY-induced immune organ atrophy, improve immune response by promoting the phosphorylation of key proteins in the MAPK signaling pathway, and promote the secretion and expression of inflammatory factors. In addition, COP1 intervention promoted SCFA production, SIgA secretion, and TJ protein expression; regulated gut microbiota; and improved intestinal barrier function. These results suggest that COP1 may be a potential food for regulating immune function, which provides a theoretical basis for the application of polysaccharides from *Chimonanthus nitens Oliv* leaves in fortified foods and the further development and utilization of *Chimonanthus nitens Oliv* leaves.

## Figures and Tables

**Table 1:** Average body weight of mice.

Day		Groups					
		NC	MC	PC	LCOP1	MCOP1	HCOP1
Body weight (g)	1	23.10 ± 0.58^Aa^	22.98 ± 0.31^Aa^	23.00 ± 0.64^Aa^	22.95 ± 0.53^Aa^	22.92 ± 0.24^Aa^	23.03 ± 0.22^Aa^
	5	26.28 ± 0.66^Aa^	24.35 ± 0.26^Cd^	24.7 ± 0.77^Ccd^	24.77 ± 0.53^Ccd^	25.7 ± 0.19^ABab^	25.28 ± 0.39^BCbc^
	9	27.7 ± 0.70^Aa^	23.35 ± 0.46^Fd^	24.65 ± 0.87^Dc^	24.95 ± 0.69^CDc^	26.38 ± 0.21^Bb^	25.93 ± 0.45^BCb^
	13	26.95 ± 0.68^ABa^	22.17 ± 0.62^Dd^	24.65 ± 0.96^Cc^	25.95 ± 0.83^Bb^	27.76 ± 0.56^Aa^	27.31 ± 0.46^Aa^
	17	28.73 ± 0.77^Aa^	20.62 ± 0.90^De^	23.62 ± 1.09^Cd^	25.4 ± 0.79^Bc^	27.53 ± 0.69^Ab^	27.78 ± 0.61^Aab^

Note: the values were presented as mean ± SD (*n* = 6). On each measure, the difference in letters between the groups indicated significant differences by the letters abcd (*p* < 0.05) and ABCD (*p* < 0.01). Duncan analysis was used for data variance analysis.

**Table 2:** Effect of COP1 on the organ indexes of mice.

Groups	Spleen index (%)	Thymus index (%)
NC	3.468 ± 0.274^Cb^	2.155 ± 0.289^Aa^
MC	1.262 ± 0.122^Ed^	0.434 ± 0.063^Cb^
PC	4.267 ± 0.082^Aa^	2.029 ± 0.310^Aa^
L-COP1	1.785 ± 0.073^Dc^	0.850 ± 0.177^Bb^
M-COP1	3.897 ± 0.152^Ba^	1.883 ± 0.204^Aa^
H-COP1	3.915 ± 0.461^Ba^	2.208 ± 0.349^Aa^

Note: the values were presented as mean ± SD (*n* = 6). On each measure, the difference in letters between the groups indicated significant differences by the letters abcd (*p* < 0.05) and ABCD (*p* < 0.01). Duncan analysis was used for data variance analysis.

## Data Availability

All data used to support the findings of this study are available from the corresponding author upon request.

## Abbreviations

COP1: *Chimonanthus nitens Oliv* polysaccharides
CY: Cyclophosphamide
MAPKs: Mitogen-activated protein kinases
SCFAs: Short-chain fatty acids
TJ: Tight junction
SIgA: Secretory immunoglobulin A
NC: The normal group
MC: The model group
PC: The positive group
LCOP1: The low-dose COP1 group
MCOP1: The medium-dose COP1 group
HCOP1: The high-dose COP1 group
H&E: Hematoxylin and eosin
PBS: Phosphate buffer solution
RT-qPCR: Real-time quantitative polymerase chain reaction
PVDF: Polyvinylidene difluoride membrane
ECL: Enhanced tetrahydrochloric acid chemiluminescence
OTUs: Operational taxonomic units
PCA: Principal component analysis
LEfSe: Linear discriminant analysis effect size
LDA: Linear discriminant analysis.

## Supplementary Materials

Supplementary Materials Table S1: dilution ratio of all primary and secondary antibodies. Figure S1: graphic abstract of this article.
