# Supplementary material for: Chimonanthus nitens Oliv Polysaccharides Modulate Immunity and Gut Microbiota in Immunocompromised Mice
Source: Oxid Med Cell Longev. 2023 Feb 15;2023:6208680. doi: 10.1155/2023/6208680 (PMC9946750; doi:10.1155/2023/6208680)
Supplement: Supplementary Materials — Table S1: dilution ratio of all primary and secondary antibodies. Figure S1: graphic abstract of this article. [file 6208680.f1.zip › Table S1.docx]

Table S1 Dilution ratio of each antibody

|  | Antibody name | Dilution ratio |
| --- | --- | --- |
| Primary antibody | Occludin1 | 1:500 |
|  | Claudin1 | 1:4000 |
|  | ZO1 | 1:5000 |
|  | ERK | 1:1000 |
|  | JNK | 1:10000 |
|  | P38 | 1:1000 |
|  | p-ERK | 1:4000 |
|  | p-JNK | 1:1500 |
|  | p-P38 | 1:1200 |
|  | GPADH | 1:200000 |
| Secondary antibody | HRP-conjugated Affinipure Goat Anti-Mouse IgG(H+L) | 1:10000 |
|  | HRP-conjugated Affinipure Goat Anti-Rabbit IgG(H+L) | 1:10000 |
