# Supplementary figures and images for: Chimonanthus nitens Oliv Polysaccharides Modulate Immunity and Gut Microbiota in Immunocompromised Mice
Source: Oxid Med Cell Longev. 2023 Feb 15;2023:6208680. doi: 10.1155/2023/6208680 (PMC9946750; doi:10.1155/2023/6208680)

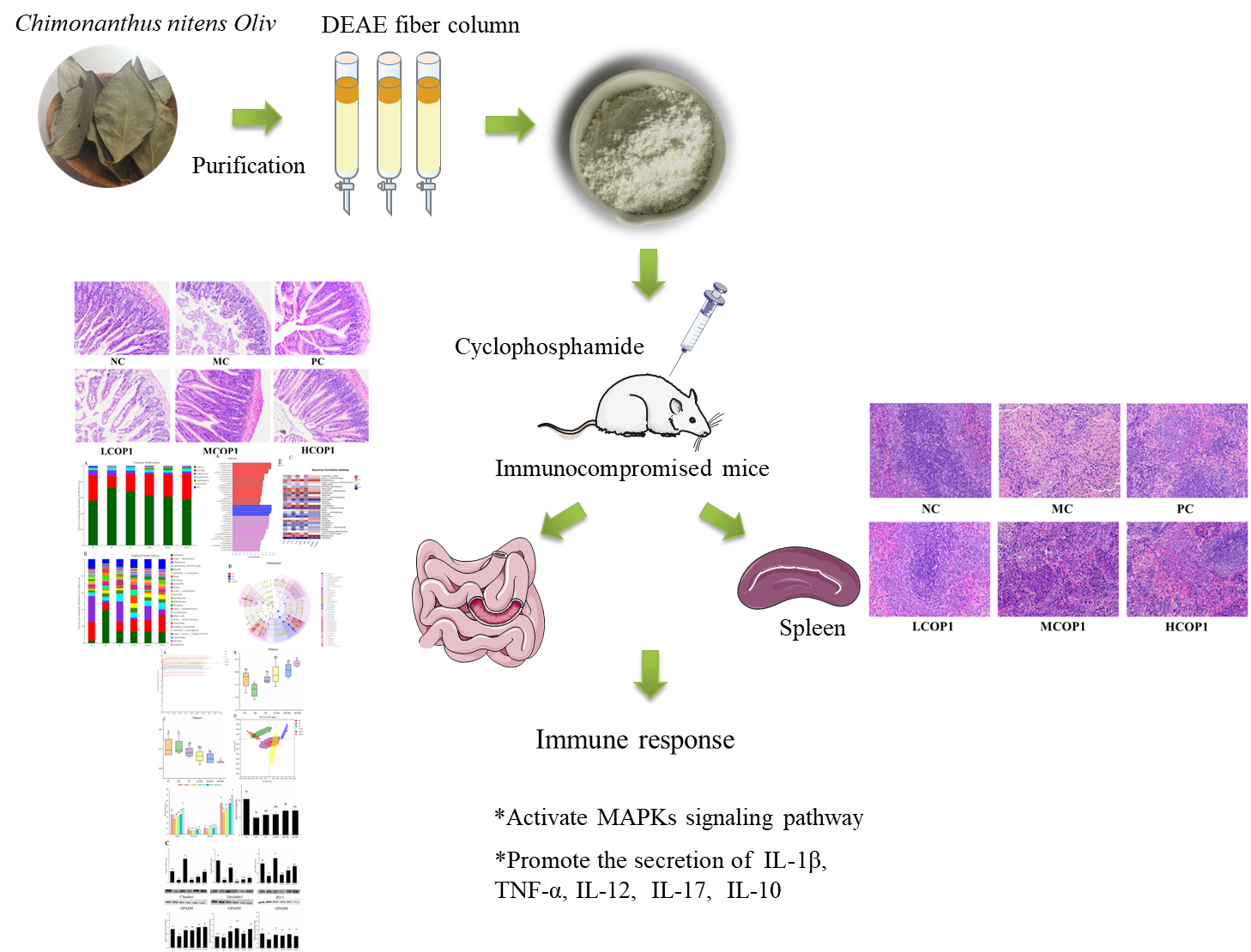


Figure S1 Graphic abstract

Supplement: Supplementary Materials — Table S1: dilution ratio of all primary and secondary antibodies. Figure S1: graphic abstract of this article. [file 6208680.f1.zip › Figure S1.docx]
